# LRG1 Promotes ECM Integrity by Activating the TGF-β Signaling Pathway in Fibroblasts

**DOI:** 10.3390/ijms241512445

**Published:** 2023-08-04

**Authors:** Han Na Park, Min Ji Song, Young Eun Choi, Dong Hun Lee, Jin Ho Chung, Seung-Taek Lee

**Affiliations:** 1 Department of Biochemistry, College of Life Science and Biotechnology, Yonsei University, Seoul 03722, Republic of Korea; qkrgkssk1024@naver.com (H.N.P.); choii03271452@gmail.com (Y.E.C.); 2 Department of Dermatology, Seoul National University College of Medicine, Seoul 03080, Republic of Korea; minjisong@snu.ac.kr (M.J.S.); ivymed27@snu.ac.kr (D.H.L.); jhchung@snu.ac.kr (J.H.C.); 3 Laboratory of Cutaneous Aging Research, Biomedical Research Institute, Seoul National University Hospital, Seoul 03080, Republic of Korea; 4 Institute of Human-Environment Interface Biology, Seoul National University, Seoul 03080, Republic of Korea; 5 Institute on Aging, Seoul National University, Seoul 03080, Republic of Korea

**Keywords:** LRG1, TGF-β, skin, aging, collagen, MMP-1, extracellular matrix, fibroblast

## Abstract

Leucine-rich alpha-2-glycoprotein 1 (LRG1) mediates skin repair and fibrosis by stimulating the transforming growth factor-beta (TGF-β) signaling pathway. In the present study, we investigated the effect of LRG1 on extracellular matrix (ECM) integrity in fibroblasts, as well as on skin aging. The treatment of dermal fibroblasts with purified recombinant human LRG1 increased type I collagen secretion and decreased matrix metalloproteinase-1 secretion. Additionally, LRG1 promoted SMAD2/SMAD3 phosphorylation in a pattern similar to that of TGF-β1 treatment. An inhibitor of TGF-β receptor 1 abolished LRG1-induced SMAD2 phosphorylation. RNA sequencing identified “extracellular region”, “extracellular space”, and “extracellular matrix” as the main Gene Ontology terms in the differentially expressed genes of fibroblasts treated with or without LRG1. LRG1 increased TGF-β1 mRNA levels, suggesting that LRG1 partially transactivates the expression of TGF-β1. Furthermore, an increased expression of type I collagen was also observed in fibroblasts grown in three-dimensional cultures on a collagen gel mimicking the dermis. LRG1 mRNA and protein levels were significantly reduced in elderly human skin tissues with weakened ECM integrity compared to in young human skin tissues. Taken together, our results suggest that LRG1 could retard skin aging by activating the TGF-β signaling pathway, increasing ECM deposition while decreasing its degradation.

## 1. Introduction

Leucine-rich alpha-2-glycoprotein 1 (LRG1) is a secreted glycoprotein that belongs to the leucine-rich repeat protein family [1]. LRG1 was initially extracted from human serum, and the subsequent cleavage of its N-terminal signaling peptide resulted in the release of a 50-kDa matrix protein into the extracellular space [2]. LRG1 is expressed in various tissues, such as the liver, lungs, kidneys, and skin. Notably, in the skin, LRG1 is expressed in fibroblasts, keratinocytes, and endothelial cells [3,4,5,6]. 

LRG1 has been extensively studied over the past few decades as a contributing factor to normal physiological and pathological responses [7,8]. In a normal physiological context, LRG1 is associated with the regulation of the immune response, and it plays a pivotal role in tissue regeneration and neovascularization. Under pathological conditions, LRG1 has been associated with inflammation, fibrosis, and cardiovascular disease. Mechanistically, LRG1 acts principally through the transforming growth factor-beta (TGF-β) signaling pathway [7].

In fibroblasts, TGF-β activates the expression of extracellular matrix (ECM) genes, particularly type I collagen, and it acts as a potent activator of matrix synthesis [9,10]. The TGF-β signaling pathway is initiated by the binding of TGF-β to TGF-β receptor 2 (TGFβR2), which subsequently activates TGF-β receptor 1 (TGFβR1) [11]. The transcription of genes involved in ECM production, such as type I collagen, is then regulated by the activation of cytoplasmic receptor-regulated SMADs (R-SMADs), namely, SMAD2 and SMAD3 [12,13,14].

Previous studies have reported on the diversity of signaling pathways induced by LRG1 in various cell types [1,7]. The ability of LRG1 to induce TGF-β signaling depends on the cell type and microenvironment. For example, in endothelial cells, LRG1 interacts with endoglin, leading to the induction of pathological angiogenesis and vascular dysfunction via the TGFβR2-activin A receptor-like type 1 (ACVRL1)-SMAD1/5/8 pathway [5,7]. Conversely, during fibrosis, LRG1 facilitates the differentiation into matrix-producing myofibroblasts via the TGFβR2-TGFβR1-SMAD2/3 pathway independently of endoglin [7]. In particular, given its independent interaction with TGFβR1, LRG1 could act as an upstream regulator of TGF-β signaling and a potential therapeutic target [2,5,7].

ECM proliferation is characterized by the upregulation of collagen types I, II, and III, and it is a prominent feature of fibrosis [15,16]. The involvement of LRG1 in fibrosis has been documented in the heart, skin, and lungs [6,17,18]. The function to promote fibrosis depends on the ability of LRG1 to activate the TGF-β and downstream signaling pathways, such as TGF-β1/SMAD, thereby inducing the synthesis of ECM proteins, including collagens [19,20,21].

Based on this evidence, we investigated the role of LRG1 in ECM integrity and skin aging. To this end, we first analyzed the LRG1-mediated expression of type I collagen and matrix metalloproteinase-1 (MMP-1), as well as the underlying molecular mechanism. Then, differentially expressed genes (DEGs) and Gene Ontologies (GOs) influenced by LRG1 were analyzed in fibroblasts. Furthermore, the effect of LRG1 on the biosynthesis of type I collagen was verified in fibroblasts cultured in a three-dimensional (3D) collagen matrix. Finally, the expression of LRG1 was analyzed in young and elderly human skin tissues. Based on these data, we propose a role for LRG1 in ECM integrity and skin aging.

## 2. Results

### 2.1. LRG1 Increases Type I Collagen Expression and Decreases MMP-1 Expression in Fibroblasts

**Figure 1:**
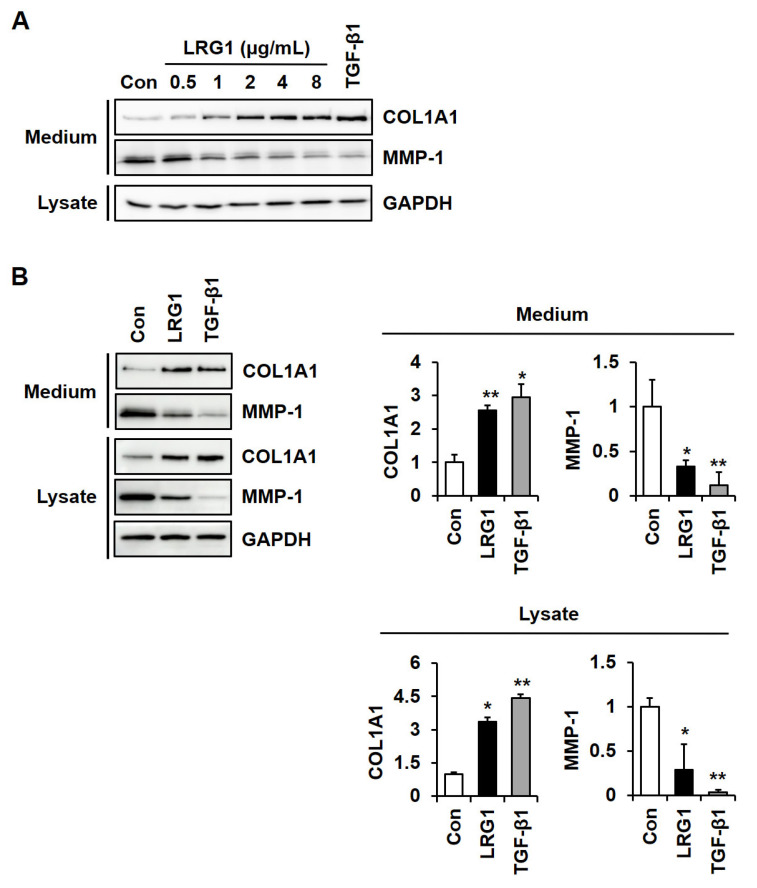
Effect of leucine-rich alpha-2-glycoprotein 1 (LRG1) on the secretion of type I collagen and MMP-1 in fibroblasts. (**A**) Type I collagen (COL1A1) and matrix metalloproteinase-1 (MMP-1) levels in human foreskin fibroblasts incubated in serum-free Dulbecco’s modified Eagle’s medium (DMEM) containing 0–8 µg/mL LRG1 or 3 ng/mL transforming growth factor-beta 1 (TGF-β1) for 24 h. (**B**) COL1A1 and MMP-1 levels in foreskin fibroblasts incubated with serum-free DMEM in the absence (Con) or presence of LRG1 (2 µg/mL) or TGF-β1 (3 ng/mL) for 24 h. Protein levels in cell lysates and conditioned media were evaluated using antibodies against pro-COL1A1, MMP-1, and glyceraldehyde 3-phosphate dehydrogenase (GAPDH) as a control. Graphs show the levels of type I collagen and MMP-1 quantified using ImageJ software (version 1.53u1), relative to the levels in non-treated cells. Each value represents the mean ± SD of three independent experiments. * *p* < 0.05 and ** *p* < 0.01 vs. Con.

To analyze the role of LRG1 in ECM integrity, we investigated the effect of human LRG1 on the levels of type I collagen and MMP-1 in foreskin fibroblasts. Recombinant human LRG1 was expressed by transfecting the human LRG1-His expression vector into HEK 293 cells, and then it was purified from the conditioned medium of the transfected cells using Ni^2+^-NTA resins (Figure S1). Treatment with LRG1 (0–8 μg/mL) led to an increase in the secretion of type I collagen and a decrease in the secretion of MMP-1 in a dose-dependent manner (Figure 1A). The same effect was observed with TGF-β1 (3 ng/mL) when used as a positive control.

Treatment with LRG1 at 2 μg/mL resulted in a statistically significant increase in type I collagen and decrease in MMP-1 in conditioned media and in cell lysates (Figure 1B). These results are comparable to those obtained with TGF-β1.

**Figure 2:**
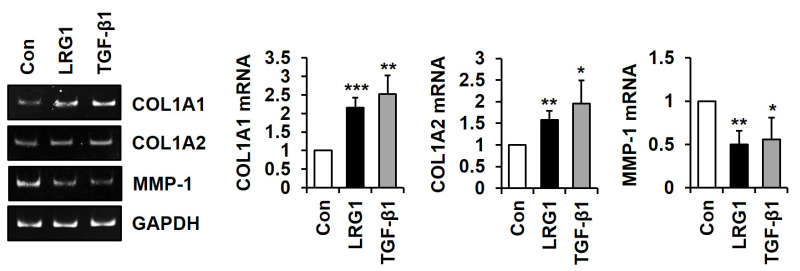
Effect of LRG1 on mRNA levels of type I collagen and MMP-1 in fibroblasts. Conventional (**left**) and quantitative (**right**) reverse transcription–polymerase chain reaction (RT-PCR) results showing relative levels of COL1A1, COL1A2, and MMP-1 mRNAs. GAPDH was used as control. Serum-starved foreskin fibroblasts were incubated without (Con) or with LRG1 (2 µg/mL) or TGF-β1 (3 ng/mL) for 12 h. Each value represents the mean ± SD of five independent experiments. * *p* < 0.05, ** *p* < 0.01, *** *p* < 0.001 vs. Con.

We examined whether the modulation of type I collagen and MMP-1 secretion by LRG1 resulted from transcriptional regulation. Conventional and quantitative reverse transcription–polymerase chain reaction (RT-PCR) showed that LRG1 significantly increased COL1A1 and COL1A2 mRNA levels while decreasing MMP-1 mRNA levels (Figure 2), suggesting that LRG1 transactivates COL1A1 and COL1A2 transcription and suppresses MMP-1 transcription. 

### 2.2. LRG1 Upregulates the Expression of R-SMADs by Activating TGF-β Receptors in Fibroblasts

**Figure 3:**
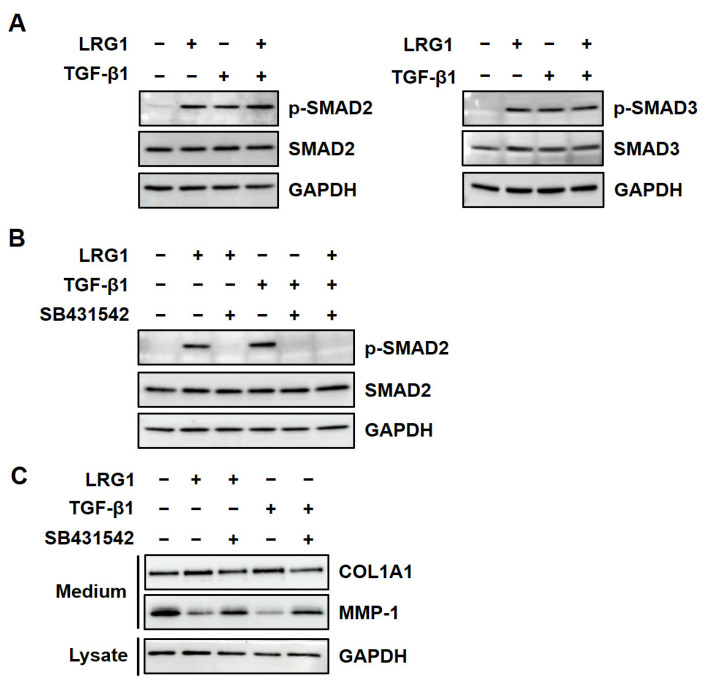
Effect of R-SMAD activation by LRG1 in fibroblasts. (**A**) Phospho-SMAD2 (p-SMAD2), SMAD2, phospho-SMAD3 (p-SMAD3), and SMAD3 levels in the lysates obtained from foreskin fibroblasts incubated with LRG1 (2 µg/mL) or TGF-β1 (3 ng/mL) for 30 min. (**B**) P-SMAD2 and SMAD2 levels in the lysates obtained from foreskin fibroblasts preincubated with SB431542 (10 µM) for 10 min and then stimulated with LRG1 (2 µg/mL) or TGF-β1 (3 ng/mL) for 30 min. (**C**) COL1A1 and MMP-1 levels in conditioned media and cell lysates obtained from foreskin fibroblasts stimulated with LRG1 (2 µg/mL) or TGF-β1 (3 ng/mL) in the presence of SB431542 (10 µM) for 24 h. GAPDH was used as control.

To determine whether TGF-β signaling was involved in LRG1-mediated type I collagen and MMP-1 secretion, we analyzed the activation of SMAD2 and SMAD3, two representative R-SMADs, in the LRG1-treated foreskin fibroblasts. The addition of LRG1 (2 μg/mL) as well as TGF-β1 (3 ng/mL) increased the phosphorylation of SMAD2 and SMAD3 (Figure 3A). 

Next, we analyzed the effect of SB431542, an inhibitor of TGFβR1, on LRG1-induced SMAD2 activation. SB431542 abolished LRG1- and TGF-β1-induced SMAD2 phosphorylation (Figure 3B). In addition, it also inhibited LRG1-mediated type I collagen secretion while restoring MMP-1 secretion (Figure 3C). These results suggest that LRG1 induced type I collagen expression through the activation of the TGF-β receptor and R-SMADs.

**Figure 4:**
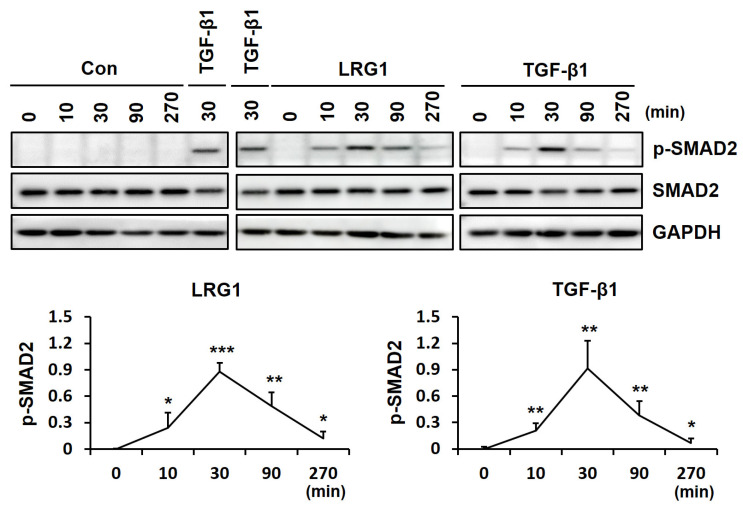
Time-dependent effect of LRG1 on SMAD2 phosphorylation in fibroblasts. Representative Western blotting images (upper panel) and relative intensity (lower panels) of p-SMAD2 in foreskin fibroblasts treated with vehicle (Con), LRG1 (2 µg/mL), or TGF-β1 (3 ng/mL) for the indicated time periods (0, 10, 30, 90, and 270 min). The relative intensity of p-SMAD2 was normalized to that of GAPDH. Each value represents the mean ± SD of five independent experiments. * *p* < 0.05, ** *p* < 0.01, *** *p* < 0.001 vs. 0 min.

To analyze the kinetics of SMAD2 activation by LRG1 and TGF-β1, we performed a time-course analysis of SMAD2 phosphorylation after treatment with LRG1 or TGF-β1 for 0–270 min in fibroblasts. The phosphorylation of SMAD2 peaked 30 min after the LRG1 treatment, the same as with the TGF-β1 treatment (Figure 4). These results suggest that LRG1 activates the TGF-β signaling pathway via the same mechanism as TGF-β1.

### 2.3. LRG1 Induces the Differential Expression of ECM-Related Genes in Fibroblasts

**Figure 5:**
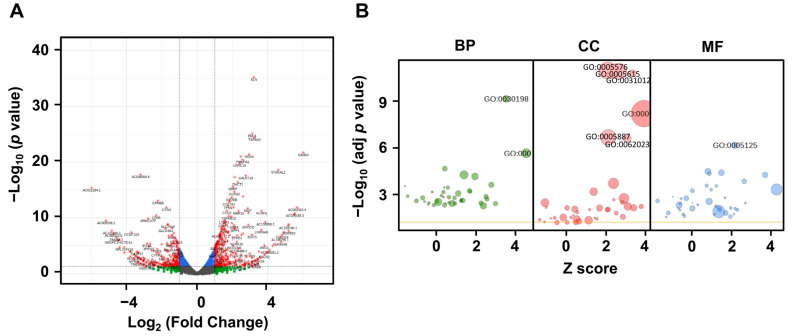
Gene Ontology (GO) enrichment analysis of differentially expressed genes (DEGs) identified in fibroblasts treated with LRG1. (**A**) DEGs identified utilizing RNA sequencing using three independent sets of RNA mixtures obtained from foreskin fibroblasts incubated either without (Con) or with LRG1 (2 µg/mL) for 12 h. DEGs, shown in red, were selected based on log_2_ (Fold Change) ≥1.0 or ≤−1.0 and *p* < 0.05. (**B**) Classification of DEGs according to GO terms belonging to biological process (BP), cellular component (CC), and molecular function (MF) categories. The significance of GO terms in each category is indicated by −log_10_ (adjusted *p* value).

To identify LRG1-modulated genes other than type I collagen and MMP-1, RNA sequencing (RNA-seq) was performed on the foreskin fibroblasts treated with or without LRG1. Among the 60,676 transcripts detected, 649 were identified as DEGs (Figure 5A). The most significant GO terms associated with these DEGs were “extracellular region” (GO:0005576; *p* = 6.8 × 10^−12^), “extracellular space” (GO:0005615; *p* = 7.9 × 10^−12^), and “extracellular matrix” (GO:0031012; *p* = 1.0 × 10^−10^). All the above GO terms belonged to the category of cellular components (Figure 5B).

**Figure 6:**
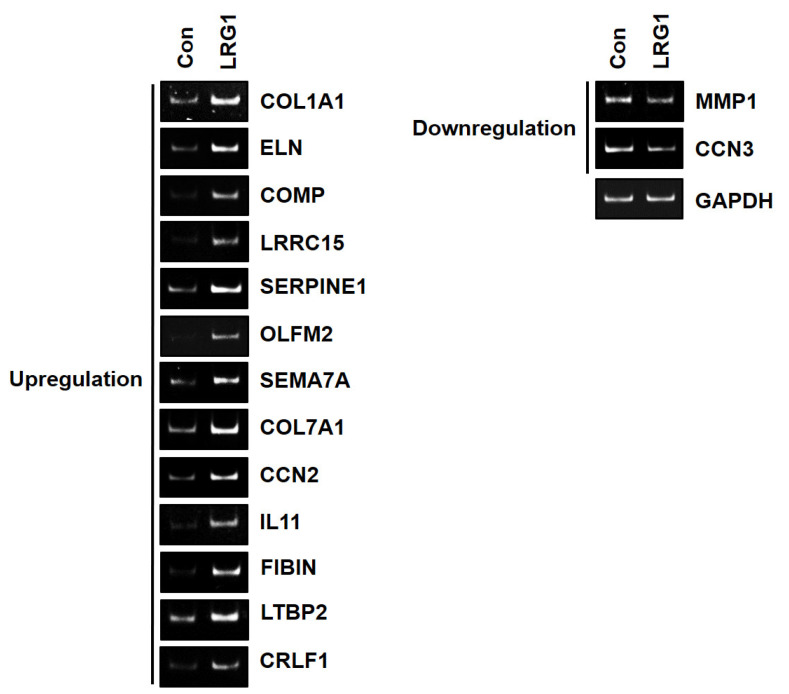
Verification of selected DEGs using RT-PCR in fibroblasts treated with LRG1. Conventional RT-PCR results showing the mRNA levels of 16 genes, including 12 upregulated genes (ELN, COMP, LRRC15, SERPINE1, OLFM2, SEMA7A, COL7A1, CCN2, IL11, FIBIN, LTBP2, and CRLF1) and 1 downregulated gene (CCN3), as well as COL1A1 and MMP-1. GAPDH was used as control. Serum-starved foreskin fibroblasts were incubated without (Con) or with LRG1 (2 µg/mL) for 12 h.

To confirm whether the genes within these three GO terms were modulated by LRG1, we selected genes with log_2_ (Fold Change) ≥1.5 or ≤−1.5. According to this criterion, we identified twelve upregulated genes (ELN, COMP, LRRC15, SERPINE1, OLFM2, SEMA7A, COL7A1, CCN2, IL11, FIBIN, LTBP2, and CRLF1) and one downregulated gene (CCN3) (Table S2). The mRNA levels of these genes in the foreskin fibroblasts treated with or without LRG1 were analyzed using RT-PCR, which confirmed the trend observed with RNA-seq (Figure 6). We included the COL1A1 and MMP-1 genes as controls for the upregulated and downregulated genes, respectively, even though they did not meet the selection criterion (Table S2). As expected, the RT-PCR results demonstrated the upregulation of COL1A1 mRNA expression and the downregulation of MMP-1 mRNA expression, confirming our hypothesis (Figure 6).

### 2.4. LRG1 Induces TGF-β Family Members in Fibroblasts

**Figure 7:**
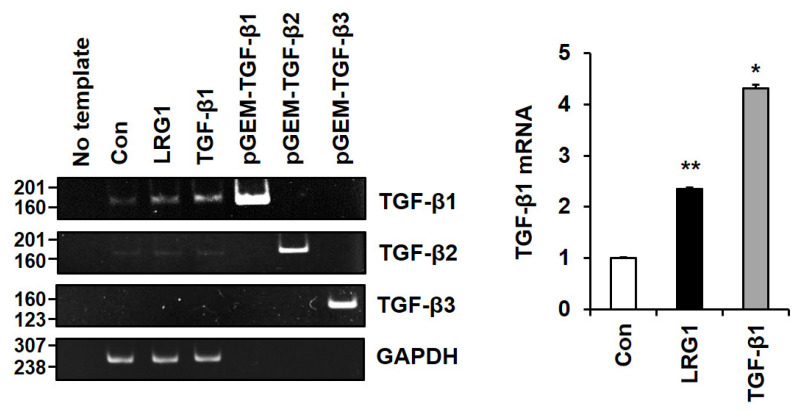
Analysis of mRNA levels of TGF-β family members in fibroblasts treated with LRG1. Conventional (**left**) and quantitative (**right**) RT-PCR results showing TGF-β1, TGF-β2, and TGF-β3 mRNA levels in foreskin fibroblasts incubated with vehicle (Con), LRG1 (2 µg/mL), or TGF-β1 (3 ng/mL) for 12 h. GAPDH was used as control, while 1 ng of pGEM-TGF-β1, TGF-β2, and TGF-β3 [22] was used as positive control for conventional RT-PCR analysis. PCR products of TGF-β1, TGF-β2, and TGF-β3 were long 161 bp, 162 bp, and 134 bp, respectively [22]. The graph on the right shows the relative level of TGF-β1 mRNA normalized to that of GAPDH. Each value represents the mean ± SD of four independent experiments. * *p* < 0.05 and ** *p* < 0.01 vs. Con.

Intriguingly, the DEGs in the foreskin fibroblasts treated with LRG1 included TGFB1, which exhibited significant upregulation (log_2_ (Fold Change) = 1.04) (Table S3). To validate this finding, the mRNA expression of TGF-β family members was analyzed in the foreskin fibroblasts treated with or without LRG1. Conventional RT-PCR revealed a clear increase in TGF-β1 mRNA levels upon LRG1 and TGF-β1 treatment, but only a faint increase in TGF-β2 mRNA levels and no TGF-β3 mRNA levels (Figure 7). Additionally, quantitative RT-PCR demonstrated that the level of TGF-β1 mRNA was significantly increased following both LRG1 and TGF-β1 treatment (Figure 7). Furthermore, a Western blot analysis showed an increase in the precursor form of TGF-β1 in the conditioned media and the cell lysates of the LRG1- and TGF-β1-treated fibroblasts (Figure S2). This finding indicates a positive feedback induction of TGF-β1 transcription, triggered by the activation of the TGF-β signaling pathway in fibroblasts.

### 2.5. LRG1 Increases the Production of Type I Collagen in Fibroblast 3D Cultures

**Figure 8:**
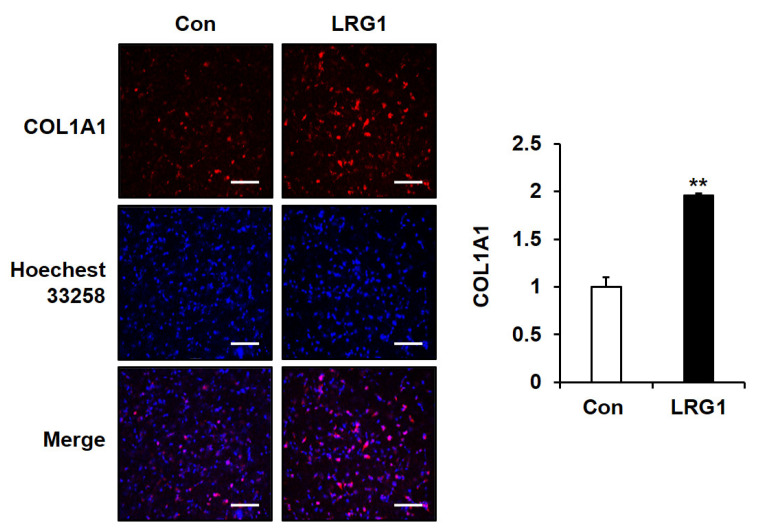
Effect of LRG1 on expression of type I collagen in a three-dimensional (3D) fibroblast culture. COL1A1 expression in foreskin fibroblasts embedded within a 3D type I collagen matrix without (Con) or with LRG1 (2 μg/mL) and incubated in serum-free DMEM for 24 h. The 3D matrix containing fibroblasts was stained with a pro-COL1A1 antibody and Rhodamine Red-X secondary antibody for type I procollagen staining, and with Hoechst 33258 for nuclear staining. The cells were analyzed using confocal fluorescence microscopy (200×). The graph to the right shows relative COL1A1 levels based on normalization to nuclear staining. Each value represents the mean ± SD of three independent experiments. ** *p* < 0.01 vs. Con. Bar = 100 µm.

To investigate the effect of LRG1 on ECM integrity under in vivo-like conditions, we assessed whether LRG1 affected the synthesis of type I collagen in foreskin fibroblasts grown in a 3D culture. LRG1 significantly increased type I collagen synthesis under the 3D culture conditions (Figure 8). 

### 2.6. LRG1 Is Downregulated in Skin Tissues during Intrinsic Aging

**Figure 9:**
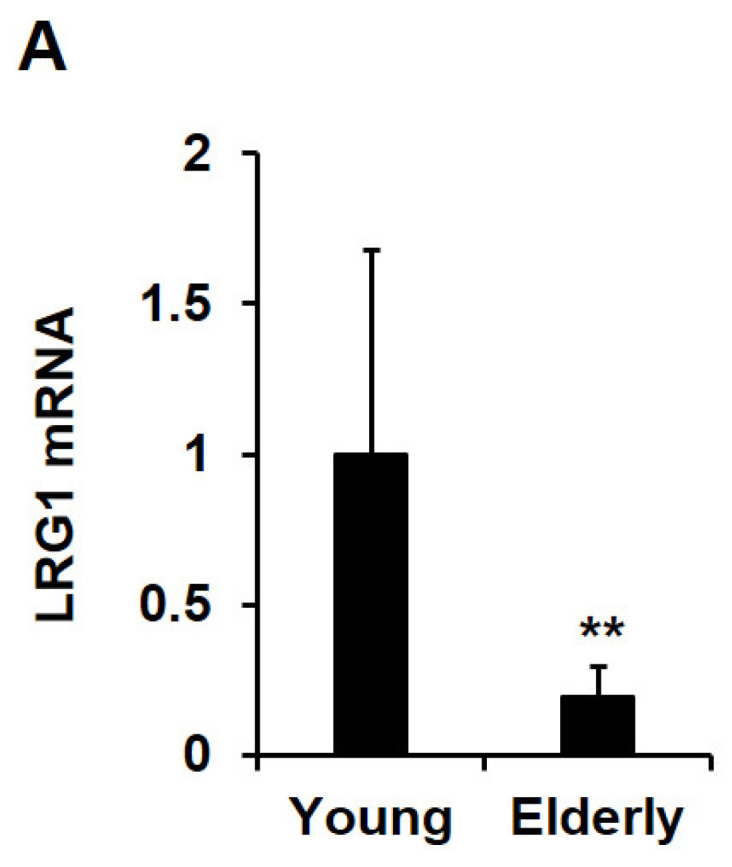

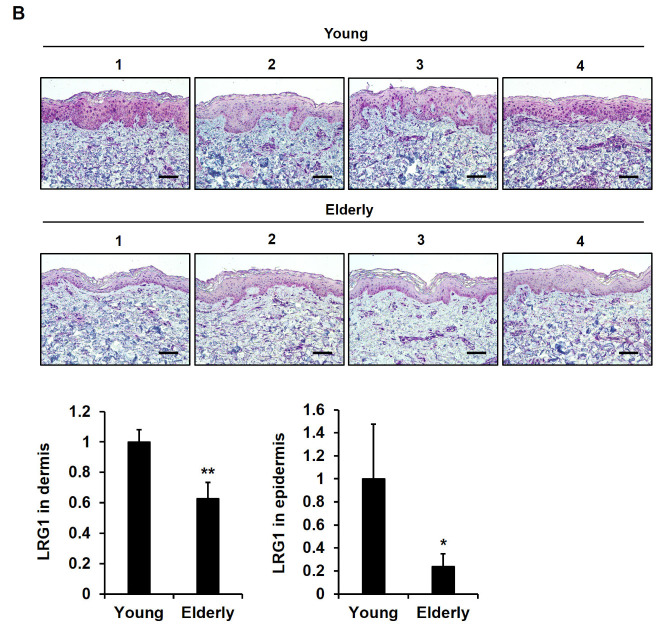
Analysis of LRG1 expression in young and elderly human skin tissues. (**A**) Quantitative RT-PCR result showing the LRG1 mRNA level in skin tissues from elderly human subjects relative to that in young human subjects (n = 9). Each value represents the mean ± SD. ** *p* < 0.01 vs. young samples. (**B**) Immunohistochemistry results of LRG1 expression in skin tissues from young (1–4: aged 20, 21, 24, and 23 years) and elderly (1–4: aged 78, 79, 80, and 76 years) human subjects. Sections were stained with anti-LRG1 antibody, horseradish peroxidase-conjugated secondary antibody, and 3-amino-9-ethylcarbazole, and they were counterstained with hematoxylin. Magnification, 200×. Bar = 100 µm. The graphs show the relative levels of LRG1 in the dermis and epidermis. Each value represents the mean ± SD of four independent experiments. * *p* < 0.05 and ** *p* < 0.01 vs. young samples.

To investigate age-related changes in LRG1 levels in human skin tissues, quantitative RT-PCR and immunohistochemistry (IHC) were conducted on sun-protected buttock skin tissues from young individuals (aged 20–30 years) and elderly individuals (aged 70–80 years). LRG1 mRNA levels were significantly lower in the skin tissues of elderly subjects than in those of young subjects (Figure 9A). The IHC staining of skin tissues from young individuals revealed a strong expression of the LRG1 protein in the epidermis, especially in the basal layer, followed by an abundant expression in the dermis (Figure 9B). Conversely, LRG1 staining was markedly lower in the skin tissues of elderly individuals (Figure 9B). Consistently, a quantitative analysis revealed a significant reduction in LRG1 expression in both the dermis and epidermis (Figure 9B).

## 3. Discussion

ECM remodeling is critical for many physiological processes, including tissue development, wound healing, and the immune response [23,24]. The dysregulation of ECM synthesis and remodeling has been associated with normal physiological conditions, such as skin aging [25], but also with pathological processes, including cancer, fibrosis, and cardiovascular diseases [26,27].

LRG1 is a glycoprotein involved in inflammation, angiogenesis, and cancer progression, as well as in the regulation of ECM synthesis and remodeling, although the precise molecular mechanisms remain unknown [2,28]. To understand how LRG1 is involved in ECM synthesis and remodeling, we analyzed the expression of type I collagen, an important component of the ECM, and MMP-1, a major ECM-degrading enzyme. In this study, we report that LRG1 increased the expression of type I collagen while decreasing MMP-1 in foreskin fibroblasts. Specifically, there was an increase in COL1A1 and COL1A2 mRNA levels along with a decrease in MMP-1 mRNA levels following LRG1 treatment, suggesting that LRG1 regulated the transcription of type I collagen and MMP-1 genes.

One of the key regulators of ECM synthesis and remodeling is the TGF-β signaling pathway. In fibroblasts, TGF-β regulates collagen synthesis and secretion, and it promotes fibroblast proliferation [11,29,30]. LRG1 has been proposed to act as a positive regulator of the TGF-β signaling pathway [5,31]. In Lewis lung cancer cells, the overexpression of LRG1 has been found to enhance the SMAD2 signaling pathway, which is also activated by TGF-β1 [32]. Additionally, a Spearman correlation analysis of IHC results identified a positive correlation between the percentages of LRG1-positive and TGF-β1-positive cells [32,33]. LRG1 has also been identified as a promoter of lung fibrosis through the modulation of TGF-β-induced SMAD2 phosphorylation and the activation of profibrotic responses in fibroblasts [6]. Consistent with these findings, we report that LRG1 induced the phosphorylation of SMAD2/3 with the same kinetics as TGF-β1. In addition, an inhibitor of TGFβR1 effectively suppressed LRG1-induced SMAD2 phosphorylation, thereby attenuating the LRG1-mediated upregulation of type I collagen and downregulation of MMP-1.

To investigate the effect of LRG1 on the regulation of genes other than those encoding type I collagen and MMP-1, RNA-seq was performed on fibroblasts treated with or without LRG1. Among the 649 DEGs detected, those with the most altered expression were related to the “extracellular region”, “extracellular space”, and “extracellular matrix” GO terms. Validation using RT-PCR confirmed the role of LRG1 in regulating the expression of ECM-related genes in the fibroblasts. 

Various mechanisms activating the TGF-β signaling pathway by LRG1 have been reported. LRG1 has been shown to bind to TGFβR2 and regulate various physiological processes involved in TGF-β signaling cascades in cancer and endothelial cells [5,33,34]. This suggests that LRG1 can directly bind to and activate TGFβRs. Moreover, LRG1 has been reported to bind to TGFβR1 and ACVRL1, thereby activating downstream mediators, such as SMADs, in the presence of TGF-β1 [5]. Additionally, LRG1 has the capability to enhance the TGF-β1 signaling pathway by binding to TGF-β1 [35,36]. Given that LRG1 interacts with both the TGF-β1/TGFβR complex and TGF-β1, it is also plausible that LRG1 can activate TGFβRs by facilitating the binding of TGF-β1 and TGFβRs.

Interestingly, the RNA-seq analysis revealed a significant increase in the expression of TGFB1 in the LRG1-treated fibroblasts. The RT-PCR analysis demonstrated that both LRG1 and TGF-β1 increased TGF-β1 mRNA levels. Therefore, we propose an alternative mechanism for the LRG1-mediated activation of the TGF-β signaling pathway, in which LRG1 facilitates the activation of TGF-β signaling by inducing TGF-β1. Previous studies have shown a positive correlation between LRG1 expression and TGF-β1 mRNA transcript levels in choroidal and retinal neovascularization models [5]. Moreover, treatment with adenovirus-delivered TGF-β1 in the carotid arteries of mice induced initial expansion through matrix accumulation, as well as TGF-β1 production [37]. These findings suggest that LRG1 can activate the TGF-β signaling pathway by inducing TGF-β1, thus resembling the positive feedback loop observed for TGF-β1. Consequently, we propose that LRG1 activates the TGF-β signaling pathway through the induction of TGF-β1, in addition to its direct or indirect binding to and activation of TGFβRs. 

It has been known that TGF-β is autoinduced through both the canonical (SMAD2/3) pathway and the non-canonical (non-SMAD2/3) pathways [38,39,40,41]. We demonstrated that both LRG1 and TGF-β1 induce TGF-β1 expression and activate SMAD2/3, resulting in an increased expression of type I collagen and a decreased expression of MMP-1. Furthermore, treatment with SB431542 led to the decreased phosphorylation of SMAD2, reduced the secretion of type I collagen, and increased the secretion of MMP-1, indicating the inhibition of the canonical TGF-β-induced signaling pathway by SB431542. However, the precise role of SB431542 in non-canonical pathways, aside from its inability to inhibit the activation of the ERK1/2 pathway, remains uncertain [38]. Consequently, while the induction of TGF-β1 by LRG1 is likely to occur via the canonical pathway, the involvement of non-canonical pathways cannot be completely ruled out.

LRG1 is overexpressed in human hypertrophic scars, promoting keratinocyte migration and skin wound healing [18,42]. Wound healing is characterized by changes in ECM integrity, and multiple cell–ECM interactions occur during the migration of fibroblasts, along with tissue formation, angiogenesis, and tissue remodeling [43].

Given that LRG1 enhances type I collagen expression and downregulates MMP-1, we analyzed whether LRG1 contributes to skin aging. Using fibroblasts cultured within a 3D collagen matrix mimicking the dermis, we demonstrated that LRG1 enhanced the expression of type I collagen. In line with the known decrease in the expression of TGF-β1 in aging skin [44], we made a similar observation in which the expression of LRG1, at both the mRNA and protein levels, was significantly lower in elderly skin tissues than in young skin tissues. These observations suggest that the downregulation of LRG1 may be associated with the disruption of collagen homeostasis and skin aging. Overall, our study provides compelling evidence for the involvement of LRG1 in the regulation of ECM dynamics in both 2D and 3D environments, and it highlights its potential as an innovative target for skin aging therapies.

## 4. Materials and Methods

### 4.1. Reagents and Antibodies

Recombinant human TGF-β1 and SB431542 were purchased from PeproTech (Princeton, NJ, USA) and Selleckchem (Houston, TX, USA), respectively. Anti-pro-COL1A1 and anti-MMP-1 antibodies were used as described previously [45]. The anti-GAPDH antibody was purchased from AbClone (Seoul, Republic of Korea). Anti-SMAD2 (SMAD family member 2), anti-phospho-SMAD2, anti-SMAD3, and anti-phospho-SMAD3 antibodies were from Cell Signaling Technology (Danvers, MA, USA). Horseradish peroxidase (HRP)-conjugated goat anti-mouse and anti-rabbit IgG antibodies were purchased from KOMA Biotech (Seoul, Republic of Korea). Rhodamine Red X-conjugated goat anti-mouse IgG (H + L) antibody was purchased from Thermo Fisher Scientific (Waltham, MA, USA).

### 4.2. Cell Culture

Primary human foreskin fibroblasts (Welgene Inc., Gyeongsan, Republic of Korea) were grown in Dulbecco’s modified Eagle’s medium (DMEM; Hyclone, South Logan, UT, USA) supplemented with 10% fetal bovine serum (Gibco/Thermo Fisher Scientific, Waltham, MA, USA). HEK 293 cells were grown in DMEM supplemented with 10% bovine serum (Gibco/Thermo Fisher Scientific), 100 U/mL penicillin, and 100 µg/mL streptomycin. All cells were grown in a humidified incubator at 37 °C in an atmosphere containing 5% CO_2_ and 95% air.

### 4.3. Construction of the hLRG1 Expression Vector

The human LRG1 expression vector pCMV-sport6-LRG1 was obtained from the Korea Human Gene Bank (Daejeon, Republic of Korea). Using this plasmid as a template, the 1085 bp DNA fragment encoding full-length human LRG1 (GenBank NM_052972.3) was amplified using PCR with a forward primer (5′-GAAGCTAGCGCTACC**ATG**TCCTCTTGGAGCAGA-3′) and a reverse primer (5′-GGGTACC**TCA***ATGATGATGATGATGATG*CTGGGACTTGGCCACTGCC-3′). These primers contained the NheI and KpnI sites for cloning (underlined letters). Bold letters indicate the start and stop codons, and italics indicate the hexahistidine tag sequence. The PCR product was digested with NheI and KpnI, and it was ligated into NheI-KpnI sites in the mammalian expression vector pcDNA3.1(+) (Invitrogen, Carlsbad, CA, USA). The ligated DNA was transfected into *Escherichia coli* XL1-Blue cells. The resulting plasmid, pcDNA3.1(+)-hLRG1-His, was confirmed via DNA sequencing.

### 4.4. Purification of Recombinant Human LRG1-His (rhLRG1) from HEK 293 Cells

Subconfluent HEK 293 cells were transfected with pcDNA3.1-hLRG1-His using the calcium phosphate method [46,47]. Stably transfected cells were selected using 1200 µg/mL G418 for two weeks and then expanded. Subconfluent HEK 293-LRG1-His cells were incubated in serum-free medium for 4.5 days. Proteins in the conditioned medium were precipitated using 80% ammonium sulfate. The resulting pellet was dissolved in phosphate-buffered saline (PBS) containing 10 mM imidazole. The resuspended sample was loaded onto a Ni^2+^-NTA agarose (QIAGEN, Hilden, Germany). Human LRG1-His was eluted using a 0.05–1 M imidazole gradient and dialyzed against PBS, as described previously [24]. 

### 4.5. Treatment of Fibroblasts with LRG1 or TGF-β1

Foreskin fibroblasts were serum-deprived for 12 h. The medium was replaced with a fresh serum-free medium, and the cells were incubated in the presence of LRG1 (2 µg/mL) or TGF-β1 (3 ng/mL) for 12 h for an mRNA analysis, or for 24 h for a protein analysis of type I collagen and MMP-1. SB431542 (10 µM) was added 10 min prior to LRG1 or TGF-β1 treatment. For the analysis of R-SMAD phosphorylation, subconfluent foreskin fibroblasts were starved in serum-free medium for 10 h, after which the medium was replaced with a fresh serum-free medium, and the cells were incubated for another 2 h. At this point, the cells were treated with either LRG1 (2 µg/mL) or TGF-β1 (3 ng/mL) for 30 min, unless otherwise specified.

### 4.6. Western Blot Analysis

Conditioned media were collected from the cultured cells via centrifugation at 2000× *g* for 5 min. The cells were washed twice with cold PBS. For the analysis of COL1A1, MMP-1, and GAPDH, the cells were lysed with a 1× sodium dodecyl sulfate (SDS) sample buffer (50 mM Tris-HCl, pH 6.8, 2% SDS, 0.1% bromophenol blue, and 10% glycerol). To analyze R-SMAD phosphorylation, the cells were lysed with a radioimmunoprecipitation assay buffer, as described previously [48], and centrifuged at 18,000× *g* for 15 min to recover the cell lysates. Protein samples were boiled for 3 min in the presence of 100 mM β-mercaptoethanol, resolved using SDS-polyacrylamide gel electrophoresis, and analyzed using Western blotting. Immunoreactive bands were detected using Immobilon Western Chemiluminescent HRP Substrate (Millipore, Bedford, MA, USA) and Amersham ImageQuant 800 (Cytiva, Marlborough, MA, USA).

### 4.7. RNA Isolation and RT-PCR Analysis

Total RNA extraction from fibroblasts, skin tissue isolation, cDNA synthesis, and RT-PCR were performed as described previously [49,50,51], except for the PCR conditions. The reaction was performed in a final volume of 10 µL, which contained 1 pM 5′ and 3′ primers, 0.2 mM dNTPs, 1× Taq PCR buffer, 50 U/mL Taq polymerase, and cDNAs synthesized from 0.15 µg total RNA. The PCR was carried out under the following conditions: denaturation at 94 °C for 30 s, annealing at the appropriate annealing temperature (Table S1) for 60 s, and extension at 72 °C for 30 s [22]. Real-time PCR was performed at the annealing temperatures described above.

### 4.8. RNA-seq and Data Analysis

RNA was sequenced using an Illumina NovaSeq6000 sequencer by Theragen Bio (Seongnam, Republic of Korea). DEGs were classified into three GO categories: biological processes, cellular components, and molecular functions. Genes whose expression was altered by LRG1 were selected if log_2_ (Fold Change) ≥1.5 or ≤−1.5. Only genes whose expression in the LRG1-treated group was above the read count mean value were selected (Table S2).

### 4.9. Biosynthesis of Type I Collagen in a 3D Culture System 

A 3D culture of fibroblasts in the collagen matrix was performed as previously described [22,52,53], with the exception of LRG1 treatment. Next, 1.5 mL of phenol red-free DMEM with or without LRG1 was added to the collagen-embedded foreskin fibroblasts, and the cells were incubated for 24 h at 37 °C in an atmosphere of 5% CO_2_ and 95% air. 

### 4.10. Acquisition of Human Skin Tissues

Punch biopsy specimens (8 mm) were obtained from the buttocks of young (aged 20–30 years) and older (aged 70–80 years) individuals without current or prior skin diseases. The skin tissues were frozen in liquid nitrogen and stored at −80 °C for an RNA analysis, or they were fixed in 10% formalin for a histological analysis. All procedures involving human subjects were approved by the Institutional Review Board of the Seoul National University Hospital (IRB No. 1410-134-621). Written informed consent was obtained from all participants, and the study was conducted in accordance with the ethical principles outlined in the Declaration of Helsinki.

### 4.11. Histological Analysis

IHC of formalin-fixed human skin tissues was performed as described previously [22] using an anti-LRG1 antibody (1:200; Proteintech, Chicago, IL, USA). The negative control was stained with a normal goat IgG antibody and showed no immunoreactivity. 

### 4.12. Statistical Analyses

All data are presented as the mean ± standard deviation (SD) of at least three independent experiments. Statistical significance was analyzed using an unpaired two-tailed Student’s *t*-test. A *p* value < 0.05 was considered statistically significant.

## 5. Conclusions

Our study provides compelling evidence of the impact of recombinant human LRG1 treatment on the expression of crucial factors involved in ECM maintenance. Specifically, LRG1 administration stimulated the expression of type I collagen while decreasing the expression of MMP-1 in human foreskin fibroblasts, both at the protein and mRNA levels. Mechanistically, LRG1 facilitated the activation of R-SMADs via the TGFβR signaling pathway, in a manner similar to that of TGF-β1. Additionally, RNA-seq revealed that LRG1 promoted the expression of genes associated with the ECM in fibroblasts. Notably, LRG1 enhanced TGF-β1 mRNA expression. Furthermore, in a 3D fibroblast culture designed to mimic the dermis, LRG1 significantly augmented type I collagen expression. Interestingly, we also noted the downregulation of both LRG1 mRNA and protein levels in elderly human skin tissues compared to their younger counterparts. This observation underscores the significant role of LRG1 in preserving the integrity of the ECM and suggests its contribution to slowing skin aging.

## Data Availability

Data are contained within the article.

## Supplementary Materials

The following supporting information can be downloaded at https://www.mdpi.com/article/10.3390/ijms241512445/s1.
