# Supplementary material for: LRG1 Promotes ECM Integrity by Activating the TGF-β Signaling Pathway in Fibroblasts
Source: Int J Mol Sci. 2023 Aug 4;24(15):12445. doi: 10.3390/ijms241512445 (PMC10418909; doi:10.3390/ijms241512445)
Supplement: Supplementary file 1 [file ijms-24-12445-s001.zip › ijms-2462808-supplementary.pdf]

Supplementary materials

LRG1 promotes ECM integrity by activating the TGF-β signaling pathway in fibroblasts

Han Na Park[1], Min Ji Song[2], Young Eun Choi[1], Dong Hun Lee[2,3,4], Jin Ho Chung[2,3,4,5], and Seung-Taek Lee[1,*]

[1] Department of Biochemistry, College of Life Science and Biotechnology, Yonsei University, Seoul 03722, Republic of Korea; qkrgkssk1024@naver.com (H.N.P.); choii03271452@gmail.com (Y.E.C)

[2] Department of Dermatology, Seoul National University College of Medicine, Seoul 03080, Republic of Korea; minjisong@snu.ac.kr (M.J.S.); ivymed27@snu.ac.kr (D.H.L.); jhchung@snu.ac.kr (J.H.C.)

[3] Laboratory of Cutaneous Aging Research, Biomedical Research Institute, Seoul National University Hospital, Seoul 03080, Republic of Korea

[4] Institute of Human-Environment Interface Biology, Seoul National University, Seoul 03080, Republic of Korea

[5] Institute on Aging, Seoul National University, Seoul 03080, Republic of Korea

* Correspondence: stlee@yonsei.ac.kr; Tel.: +82-2-2123-2703

Table S1. Primer sequences used for RT-PCR of human *LRG1* and selected DEG mRNAs.

Gene symbol	Nucleotide sequence	Nucleotide position	Annealing temp. (°C)	GenBank #
LRG1	5'-CTAGGGCAGCCAAACTGGGACA -3'<br>5'-CTGAAGCCAACGATAGAGGTCGCT-3'	870-891<br>968-945	58.5	NM_052972
ELN	5′-ACCTGGTTGACCTGTCATGGC-3′<br>5′-ACTGCTCTGAAGTTCAGTGGACC-3′	2916-2936<br>3094-3072	63	NM_ 000501
COMP	5′-ACGTGGTCTTGGACACAACCAT-3′<br>5′-AGCTGATGGGTCTCATAGTCCTCT-3′	2165-2186<br>2297-2275	63	NM_ 000095
LRRC15	5′-TCAACATCACAGTCAGCTGGCAG-3′<br>5′-TGACTGTGACTCGAGGAGGAG-3′	3365-3385<br>3539-3519	66	NM_130830
SERPINE1	5′-CAGACCTGGTTCCCACTGAGG-3′<br>5′-GCCAGTGCCACAGTGGACT-3′	1679-1699<br>1880-1862	66	NM_ 000602
OLFM2	5′-AGTATTCCCACATCTCGATGCTGG-3′<br>5′-GATCACCCTTGAGGGACACAGG-3′	1387-1410<br>1601-1580	66	NM_ 058164
SEMA7A	5′-TGAGGCCTGAGTCCTTCTGGA-3′<br>5′-ACATGCAAGGCGGCTGTCCT-3′	2423-2443<br>2582-2573	66	NM_003612
COL7A1	5′-TGGTACCATCGGGCTGTGACA-3′<br>5′-AGGGGATGCTGAATCTCAGCTCA-3′	8737-8757<br>8932-8910	63	NM_000094
CCN2	5′-CCCCAGTGACAGCTAGGATGT-3′<br>5′-TGCCACAAGCTGTCCAGTCT-3′	1643-1663<br>1798-1779	63	NM_ 001901
IL11	5′-CTGCACCTGACACTTGACTGG-3′<br>5′-CGGAAGGACTGTCTCTAACTAGG -3′	694-714<br>883-861	63	NM_000641
FIBIN	5′-TGCTTTGCATGCCTGCCAACC-3′<br>5′-CAGTGTGACTGTGCAACCACTAC-3′	1527-1547<br>1751-1729	66	NM_203371
LTBP2	5′-TGTGGTCTTGTTCAGAAGCAGCC-3′<br>5′-GGTTGGAGTGAGTCTCTGCTTGT-3′	6704-6726<br>6834-6812	63	NM_000428
CRLF1	5′-TCAGCTTCCGCCTCTACGAC-3′<br>5′-TCTGCGTCTCCACGTGGCA-3′	1291-1310<br>1457-1439	63	NM_004750
CCN3	5′-CAGCTCTGAACTTCCAAGCTCC-3′<br>5′-GGCTATGAGGGACCAGTCATCT-3′	1641-1662<br>1885-1864	63	NM_002514

Table S2. Read count datasets of RNA-seq for selected 13 DEGs and *COL1A1, MMP1,* and *GAPDH* genes.

Gene symbol	Negative control	LRG1	log_2 (Fold Change)	*p* value	*q* value
COL1A1	178,141	336,523	1.03	1.4×10^{-5}	2.0×10^{-3}
ELN	1,503	12,978	3.23	6.1×10^{-36}	1.4×10^{-31}
COMP	406	2,087	2.48	9.5×10^{-22}	3.6×10^{-18}
LRRC15	332	1,638	2.42	1.6×10^{-20}	4.4×10^{-17}
SERPINE1	6,727	27,125	2.13	4.9×10^{-18}	9.4×10^{-15}
OLFM2	216	859	2.11	2.3×10^{-15}	3.2×10^{-12}
SEMA7A	794	2,653	1.86	9.8×10^{-14}	1.1×10^{-10}
COL7A1	5,123	16,447	1.8	1.5×10^{-13}	1.6×10^{-10}
CCN2	1,230	3,672	1.7	5.2×10^{-12}	4.4×10^{-9}
IL11	162	467	1.64	1.7×10^{-9}	8.2×10^{-7}
FIBIN	159	432	1.56	1.2×10^{-8}	4.6×10^{-6}
LTBP2	12,858	34,045	1.52	2.8×10^{-10}	1.7×10^{-7}
CRLF1	1,008	2,622	1.5	1.1×10^{-9}	5.8×10^{-7}
MMP1	77,803	37,783	-0.92	1.0×10^{-4}	1.1×10^{-2}
CCN3	4,136	1,139	-1.74	1.4×10^{-12}	1.3×10^{-9}
GAPDH	83,285	68,474	-0.17	4.8×10^{-1}	1.0

Table S3. Read count datasets of RNA-seq for *TGFB* family members.

Gene symbol	Negative control	LRG1	$\log_2$ (Fold Change)	*p* value	*q* value
TGFB1	2,732	5,007	0.99	3.7×10^{-5}	4.8×10^{-36}
TGFB2	200	208	0.17	5.4×10^{-1}	1.0
TGFB3	105	71	-0.45	1.8×10^{-1}	1.0

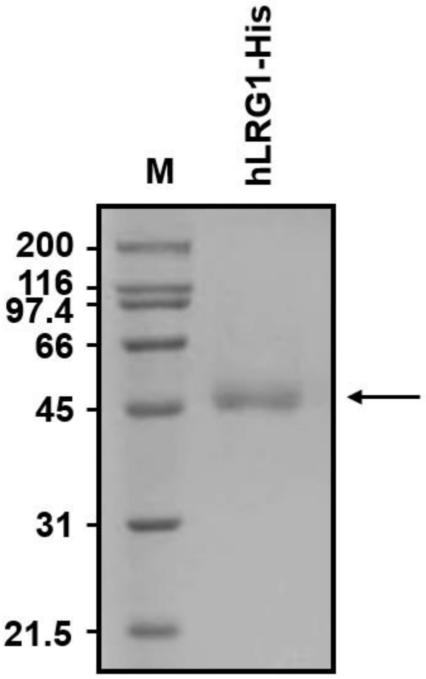

Figure S1. Purification of recombinant human LRG1-His.

The recombinant human LRG1-His polypeptide was purified from the conditioned medium of HEK 293 cells stably transfected with pcDNA3.1-hLRG1-His. The purified hLRG1-His polypeptide was resolved by SDS-PAGE and stained with Coomassie Brilliant Blue. The molecular weight marker (M) is shown on the left margin of the gel.

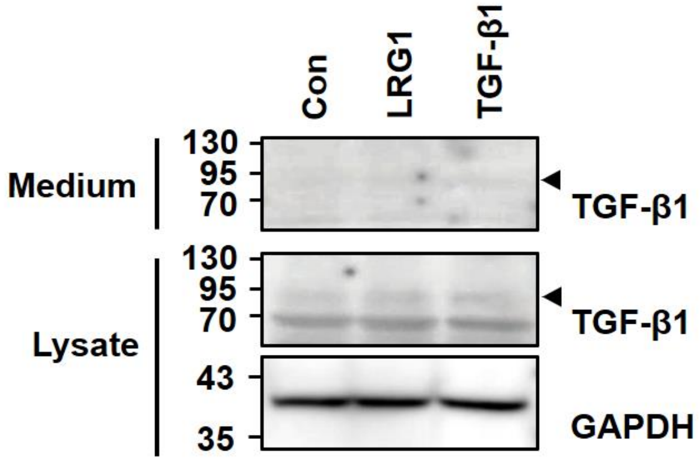

Figure S2. Analysis of TGF-β1 levels expressed in fibroblasts treated with LRG1.

Human foreskin fibroblasts were incubated in serum-free DMEM containing 2 μg/mL LRG1 or 3 ng/mL TGF-β1 for 24 h. TGF-β1 levels in conditioned media and cell lysates were analyzed in non-reduced SDS-PAGE and western blotting with an antibody against TGF-β1. GAPDH was used as loading control. The molecular weight marker is shown on the left margin of the blot. Arrowheads indicate positions for the dimer of TGF-β1 precursor.
